# Regulation of Glucose Homeostasis by KSR1 and MARK2

**DOI:** 10.1371/journal.pone.0029304

**Published:** 2011-12-19

**Authors:** Paula J. Klutho, Diane L. Costanzo-Garvey, Robert E. Lewis

**Affiliations:** Eppley Institute for Research in Cancer and Allied Diseases, University of Nebraska Medical Center, Omaha, Nebraska, United States of America; Instituto de Investigación Sanitaria INCLIVA, Spain

## Abstract

Protein scaffolds control the intensity and duration of signaling and dictate the specificity of signaling through MAP kinase pathways. KSR1 is a molecular scaffold of the Raf/MEK/ERK MAP kinase cascade that regulates the intensity and duration of ERK activation. Relative to wild-type mice, *ksr1^-/-^* mice are modestly glucose intolerant, but show a normal response to exogenous insulin. However, *ksr1^-/-^* mice also demonstrate a three-fold increase in serum insulin levels in response to a glucose challenge, suggesting a role for KSR1 in insulin secretion. The kinase MARK2 is closely related to C-TAK1, a known regulator of KSR1. Mice lacking MARK2 have an increased rate of glucose disposal in response to exogenous insulin, increased glucose tolerance, and are resistant to diet-induced obesity. *mark2^-/-^ksr1^-/-^* (DKO) mice were compared to wild type, *mark2^-/-^*, and *ksr1^-/-^* mice for their ability to regulate glucose homeostasis. Here we show that disruption of KSR1 in *mark2^-/-^* mice reverses the increased sensitivity to exogenous insulin resulting from MARK2 deletion. DKO mice respond to exogenous insulin similarly to wild type and *ksr1^-/-^* mice. These data suggest a model whereby MARK2 negatively regulates insulin sensitivity in peripheral tissue through inhibition of KSR1. Consistent with this model, we found that MARK2 binds and phosphorylates KSR1 on Ser392. Phosphorylation of Ser392 is a critical regulator of KSR1 stability, subcellular location, and ERK activation. These data reveal an unexpected role for the molecular scaffold KSR1 in insulin-regulated glucose metabolism.

## Introduction

*Par-1* (Partitioning defective) was discovered in a screen for genes that regulate cytoplasmic localization in *Caenorhabditis elegans* [1]. Mutations of *Par-1* are maternally embryonic lethal due to a failure to properly divide [2]. In addition, *Par-1* is necessary for polarization of cells in *Drosophila melanogaster*, *Xenopus laevis*, and mammals [3]–[7].

In mammals, there are four Par-1 homologs that comprise the MARK (Microtubule Affinity Regulating Kinase) family. This family consists of four closely related proteins (MARK1–4) that have been shown to play a role in cell polarity, microtubule stability, protein stability, and cell cycle control [8]. Although similar in structure, the MARK proteins have different subcellular localizations [9]–[12]. Phosphorylation of many MARK targets generates a 14-3-3 binding site [9], [11], [13]–[21]. 14-3-3 regulates the subcellular localization of many proteins (reviewed in [22]).

Two *mark2^-/-^* (EMK/Par-1b) mouse lines have been independently generated that implicate MARK2 in the regulation of immune homeostasis [23], fertility [24], learning, memory [25], growth and metabolism [26]. C-TAK1 (MARK3/Par-1a/p78) has been implicated in pancreatic [10], liver [27], and colorectal cancers [28], hippocampal function [29], and metabolism [12].

In *C. elegans* Par-1 plays a negative role in vulva induction and may function by negatively regulating the scaffolding protein KSR1 (Kinase Suppressor of Ras1) [2], [15], [30]. In mammalian cells, C-TAK1 has been shown to negatively regulate KSR1 by phosphorylation of Ser392. Phosphorylation of this site sequesters KSR1 in the cytoplasm [18], [31]. KSR1 is a molecular scaffold of the Raf/MEK/ERK MAP kinase pathway [32]–[38]. KSR1 enhances Raf-1 activity in a kinase-independent manner [35]. Upon Ras activation, KSR1 translocates to the plasma membrane, due to the activation of Protein Phosphatase 2A (PP2A). PP2A removes the phosphate from Ser392, which releases 14-3-3 from KSR1, exposing a membrane targeting sequence in the CA3 domain of KSR1[39].

*ksr1^-/-^* mice are grossly normal, however, there are subtle defects. Mice lacking KSR1 have defects in T-cell activation [38]. These mice also display defects in neuronal signaling [40]. KSR1 has been shown to play a role in oncogenesis [38], [41]. k*sr1^-/-^* mice display a decrease in tumor formation caused by polyomavirus MT or by treatment with 12-*O*-tetradecanoylphorbol-13-acetate (TPA) [38], [41].

In addition, *ksr1^-/-^* mice display enlarged adipocytes, though the fat mass is the similar to wild-type (WT) mice [42]. This indicates that these mice have fewer adipocytes, possibly indicating a role for KSR1 in adipogenesis. ERK both promotes and inhibits adipogenesis. Deletion of KSR1 prevents adipogenesis *in vitro*, and this is rescued by expression of ectopic KSR1 [43]. In addition, KSR1 levels increase through the first four days of adipogenic induction.

The interaction between KSR1 and MARK2 was first found by mass spectrometry of peptides derived from proteins associated with immunoprecipitated KSR1 [44]. We sought to confirm this interaction and to determine if MARK2 was regulating KSR1 in a manner similar to C-TAK1. We found that MARK2, like C-TAK1, phosphorylates KSR1 *in vitro*. *In vivo*, MARK2 appears to negatively regulate KSR1 in insulin sensitivity. However, deletion of KSR1 in *mark2^-/-^* animals does not effect glucose tolerance of *mark2^-/-^* animals but does increase serum insulin, suggesting a novel role for KSR1 in the regulation of insulin secretion.

## Methods

### Western blot analysis

293T cells were lysed in NP40 lysis buffer (20 mM Tris pH 8, 137 mM NaCl, 10% glycerol, 1% NP40). For western blot analysis, lysates or immunoprecipitations were resolved on an SDS-PAGE gel and transferred to a nitrocellulose membrane. Membranes were probed with primary antibody diluted in Odyssey blocking buffer: TBS + 0.2% Tween. Primary antibodies used were: anti-KSR1 (BD Transduction Laboratories), anti-FLAG (M2 Sigma), and anti-HA (Sigma). Membranes were then visualized by scanning using the Odyssey system (LI-COR). Bands were quantified using Odyssey software and values analyzed using Microsoft Excel.

### Immunoprecipitations

Immunoprecipitations were performed by incubating cellular lysates overnight at 4°C using anti-FLAG-agarose or anti-HA-agarose (Sigma). For anti-FLAG immunoprecipitations, proteins were eluted using 75 µg/mL FLAG peptide NP40 lysis buffer for 45 minutes at 4°C. The supernatant was boiled with electrophoresis sample buffer. For all other immunoprecipitations, the agarose beads were boiled with electrophoresis sample buffer. Each supernatant was then resolved by SDS-PAGE electrophoresis and transferred to a nitrocellulose membrane for analysis by western blot.

### Kinase Assay

293T cells were transfected using calcium phosphate transfection [45]. Thirty-six hours later cells were lysed in NP40 lysis buffer and immunoprecipitations performed. Immunoprecipitates were washed with kinase wash buffer (50 mM Tris pH 7.5, 120 mM NaCl, 1 mM EDTA, 0.5% NP-40) three times, then with Antarctic phosphatase buffer twice. Immunoprecipitates were treated with Antarctic phosphatase (New England Biolabs) 30 minutes at 30°C. To deactivate phosphatase activity, the reaction was incubated at 65°C for 1 hour. Immunoprecipitates were then washed three times with kinase buffer (40 mM Tris pH 7.4, 20 mM MgCl_2_, 2 mM MnCl_2_, 25 uM ATP, 0.5 mM DTT), combined and incubated at 30°C for 30 min. Reactions were terminated by the addition of electrophoresis sample buffer.

### Generation and housing of mice

*ksr1^-/-^* and *mark2^-/-^* mice were described previously [38], [43]. *mark2^-/-^ksr1^-/-^* (DKO) mice were generated by interbreeding *mark2^+/-^* and *ksr1^-/-^* mice to generate *mark2^+/-^ksr1^-/-^* mice. *mark2^+/-^ksr1^-/-^* mice were bred to generate DKO mice. The Institutional Animal Care and Use Committee (University of Nebraska Medical Center, Omaha, NE) approved all studies (permit number 05-018-03). Animals were maintained on a 12-hour light/dark schedule (light on at 0600) and had free access to laboratory chow (Harlan Teklad LM 485) and water.

### Metabolite assays

Blood glucose was measured with an Ascensia Glucometer Elite (Fisher Scientific). Plasma insulin was measured with the Mouse Insulin Elisa Kit (ChrystalChem, Chicago, IL) using mouse standards.

### Glucose and insulin tolerance tests

Insulin tolerance tests (ITT) were performed after a four hour fast on 8–10 week old mice. Blood glucose values were measured immediately before and at 15 min intervals after i.p. injection of insulin (0.30 IU/kg HumulinRinsulin). Glucose tolerance tests (GTT) were performed after a ten hour fast on 10–12 week old animals. Mice were injected i.p. with D-glucose (20% solution, 2 g/kg of body weight) and blood glucose levels determined at the indicated times.

## Results

### MARK2 interacts with KSR1

**Figure 1:**
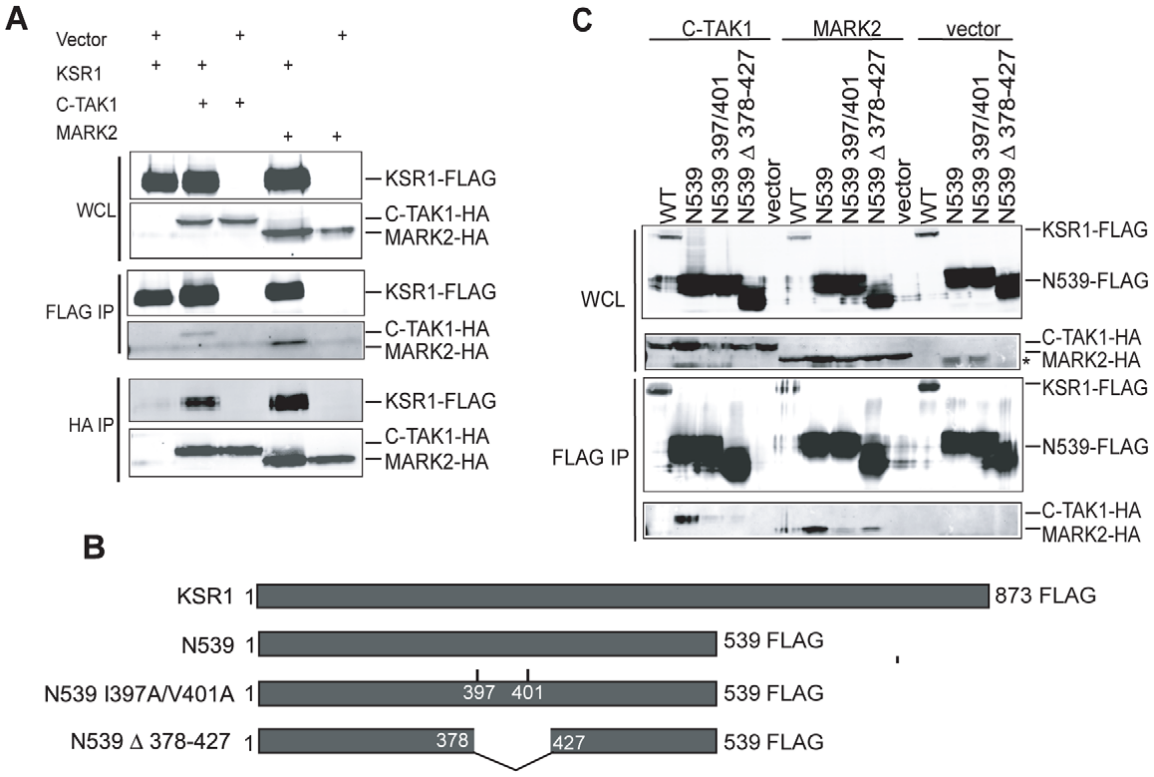
MARK2 interacts with KSR1. A. KSR1-FLAG, C-TAK1-HA, MARK2-HA and their respective vectors were transfected in combination in 293T cells. Thirty-six hours after transfection, cells were lysed and immunoprecipitated with FLAG- and HA-specific antibodies. Proteins were detected on a western blot using antibodies to each epitope tag. B. Schematic of KSR1 constructs used. C. KSR1-FLAG WT or mutants were co-transfected with C-TAK1-HA, MARK2-HA, or empty vectors and cells were lysed and immunoprecipitations performed as in A. IP: immunoprecipitation, WCL: Whole Cell Lysate. * non-specific band.

One of the mammalian homologs of Par-1, C-TAK1, negatively regulates KSR1 by binding and phosphorylating KSR1 on Ser392 [18]. C-TAK1 and MARK2 are in the MARK family of kinases, a sub-family of the AMPK family. MARK family members are highly conserved, suggesting that MARK2 may regulate KSR1 in a manner similar to C-TAK1. To test this possibility, MARK2 or C-TAK1 proteins were ectopically expressed with KSR1 in 293T cells and tested for their ability to co-immunoprecipitate with the scaffold. When KSR1 was immunoprecipitated, MARK2 and C-TAK1 were each detected in the precipitate (Fig. 1A). To confirm this interaction, immunoprecipitation of MARK2 or C-TAK1 was performed. When MARK2 or C-TAK1 was immunoprecipitated, KSR1 was detectable in the immunoprecipitation (Fig. 1A).

Mutation of Ile397 and Val401 to alanines disrupts binding to the MARK family member C-TAK1 [18]. To determine if MARK2 interaction is dependent on the same amino acids in KSR1, MARK2 was ectopically co-expressed in 293T cells with WT and mutated forms of KSR1 (Fig. 1B and 1C). A truncated form of KSR1 lacking the C-terminal kinase domain (KSR1 N539) was co-immunoprecipitated with both MARK2 and C-TAK1 (Fig. 1C). However, KSR1 N539 lacking the residues from 378–427 reduced binding to both MARK2 and C-TAK1. KSR1 N539 I397A/V401A reduced binding to MARK2 compared to WT KSR1. However, binding of C-TAK1 is completely abolished. This indicates that additional determinants on KSR1 for MARK2 association may be present.

### MARK2 phosphorylates KSR1

**Figure 2:**
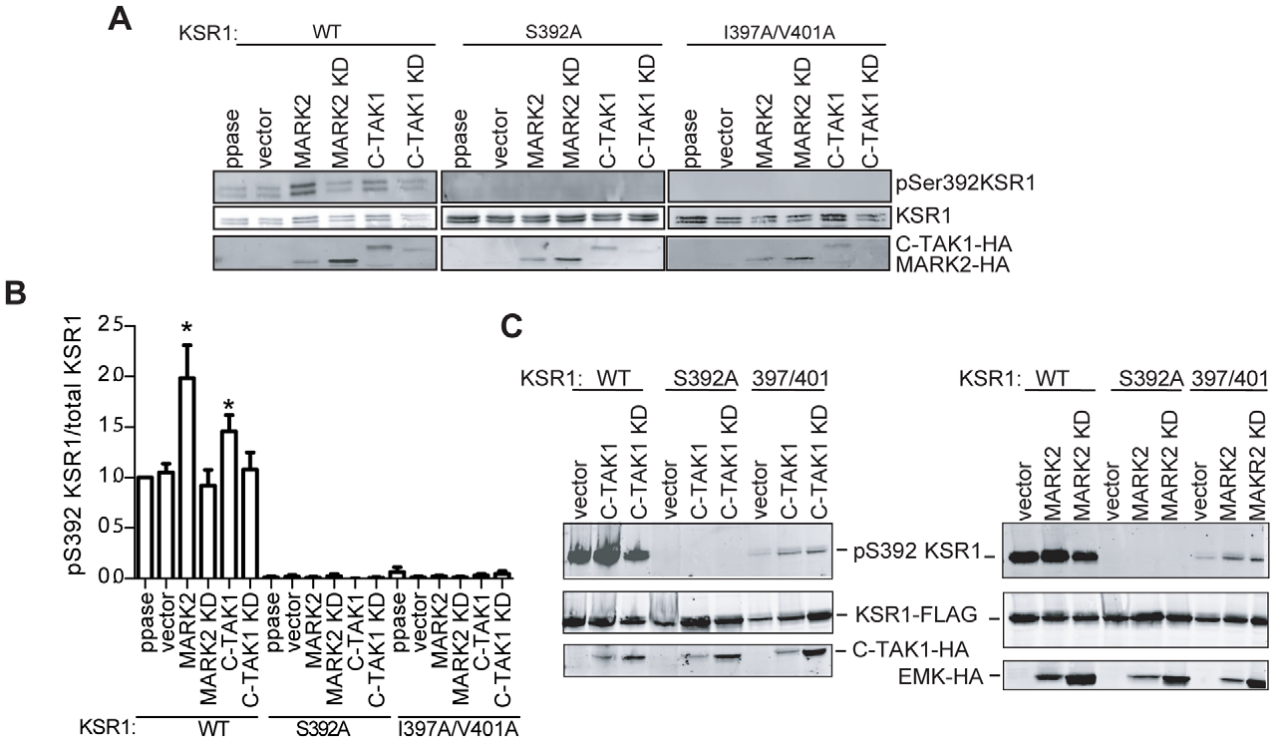
MARK2 phosphorylates KSR1 at Ser392 *in vitro* and *in vivo*. A. KSR1-FLAG, C-TAK1-HA, and MARK2-HA were individually transfected into 293T cells. Thirty-six hours later cells were lysed and HA- or FLAG-immunoprecipitations were performed. KSR1-FLAG immunoprecipitates were phosphatase treated, then incubated with MARK2-HA or C-TAK1-HA immunoprecipitates in the presence of ATP. Western blots were performed and immunoblotted with an anti-pS392 KSR1 specific antibody, anti-KSR1 antibody, or anti-HA antibody. B. Quantification of pS392 KSR1/total KSR1 from panel A, normalized to phosphatase treated WT KSR1 control. Results are the mean +/- S.D. of three independent experiments. C. KSR1-FLAG, C-TAK1-HA, MARK2-HA and their respective empty vectors were transfected in combination in 293T cells. Thirty-six hours after transfection cells were lysed. Proteins were detected on a western blot using antibodies to pS392KSR1, KSR1, or epitope tag of MARK2 and C-TAK1. *p<0.05 compared to ppase treated control.

C-TAK1 phosphorylates KSR1 at S392, forming a 14-3-3 binding site. When 14-3-3 is bound to S392, it sequesters KSR1 in the cytoplasm, away from the MAP kinase pathway [18]. To determine if MARK2 is also able to phosphorylate KSR1 an *in vitro* kinase assay was performed. When wild-type MARK2 was incubated with KSR1 in the presence of ATP, phosphorylation of Ser392 was detectable with a pSer392-specific antibody (Fig. 2A and B). No phosphorylation is detectable when Ser392 is mutated to alanine (S392A), indicating that the antibody is specific for KSR1 pSer392. A kinase-dead version of MARK2 (MARK2 KD) [46] was unable to phosphorylate Ser392. The *in vitro* assay was also performed with C-TAK1 as a positive control. MARK2 phosphorylates KSR1 *in vitro* as well as C-TAK1. In addition, both MARK2 and C-TAK1 must associate with KSR1 to phosphorylate the scaffold. KSR1 I397A/V401A, which disrupts binding to both MARK2 and C-TAK1 ([18] and Fig. 1C), is not phosphorylated on Ser392 by either MARK2 or C-TAK1 (Fig. 1C).

To confirm these results an *in vivo* kinase assay was performed. 293T cells were transfected with KSR1 alone or in combination with MARK2, MARK2 KD, C-TAK1, C-TAK1 KD, or vector control. *In vivo*, KSR1 has a high level of basal phosphorylation, presumably due to endogenous MARK family members present in 293T cells (Fig. 2C). However the amount of pS392 KSR1 increased upon the co-expression of MARK2 or C-TAK1, but not vector controls. This assay also confirms that Ile397/Val401 are important determinants on KSR1 for the ability of MARK2 and C-TAK1 to phosphorylate KSR1. These data suggest that MARK2 phosphorylates KSR1 on Ser392, which has been shown previously to function as a negative regulatory site [18].

### DKO mice are small and lean

**Figure 3:**
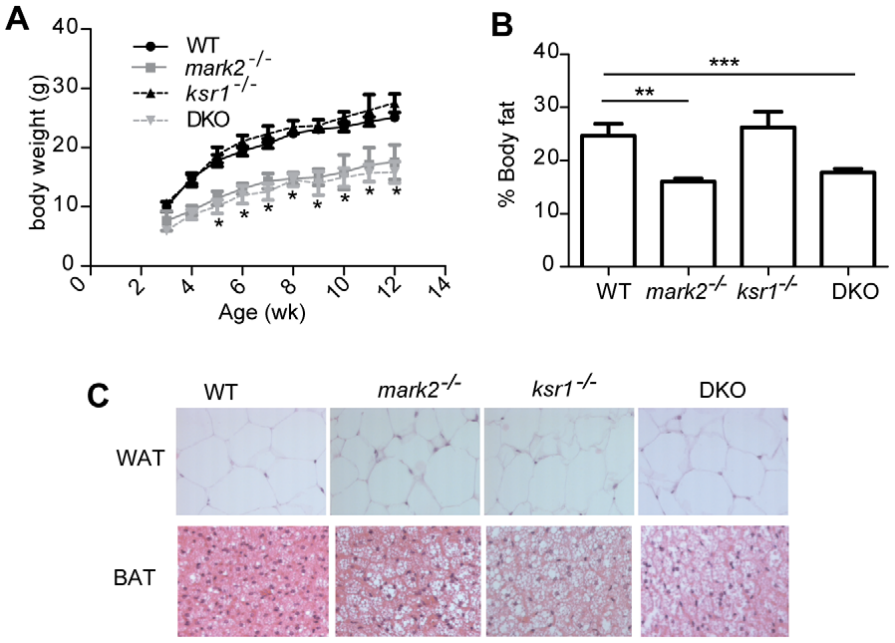
Deletion of KSR1 in *mark2^-/-^* mice does not revert their growth defect. A. Body weights of WT (black circle), *mark2^-/-^* (grey square), *ksr1^-/-^* (black triangle) and DKO mice (grey triangle) from weaning until 12 weeks of age. B. Adiposity of 12–16 week old mice, determined by DEXA. C. Hematoxylin and eosin stain of WAT and BAT from WT, *mark2* ^-/-^, *ksr1^-/-^* and DKO mice. *p<0.05 **p<0.01 ***p<0.001 compared to WT controls.

The genetic interaction between MARK2 and KSR1 was examined by the generation of *mark2* ^-/-^ *ksr1* ^-/-^ (DKO) mice. *mark2* ^-/-^ mice display dwarfism [24]. In order to determine the extent to which KSR1 contributes to the action of MARK2, a growth curve of DKO mice were generated. *mark2* ^-/-^ mice are smaller than their WT littermates at birth and remain smaller through adulthood (Fig. 3A and [26]). In contrast, *ksr1* ^-/-^mice grow at a rate similar to WT mice. DKO mice are smaller at birth than WT mice and remain small throughout life, similar in size to *mark2* ^-/-^ mice. This result may indicate that MARK2 is epistatic to KSR1 in growth regulation.

*mark2* ^-/-^ mice are also lean [26], while *ksr1* ^-/-^ mice have normal adiposity [47]. To determine if the deletion of *ksr1* ^-/-^ is able to revert the lean phenotype of *mark2* ^-/-^ mice, the percent body fat of DKO mice was determined by DEXA (Fig. 3B). DKO mice have similar adiposity to *mark2* ^-/-^ mice, and are leaner than WT and *ksr1* ^-/-^ mice. These data indicate that the deletion of KSR1 is also unable to revert the lean phenotype of *mark2^-/-^* mice.

Although *ksr1* ^-/-^ mice display a normal percent body fat, they do display an increase in adipocyte size ([43], Fig. 3B and 3C) in white adipose tissue (WAT) and brown adipose tissue (BAT). This is in contrast to *mark2* ^-/-^ mice, which, although leaner, have adipocytes similar in size to WT mice ([26] and Fig. 3B and 3C). Sections of DKO WAT and BAT indicate that the adipocytes are not enlarged (Fig. 3C).

### Disruption of KSR1 in mark2^-/-^ mice reverts insulin sensitivity but not glucose tolerance

**Figure 4:**
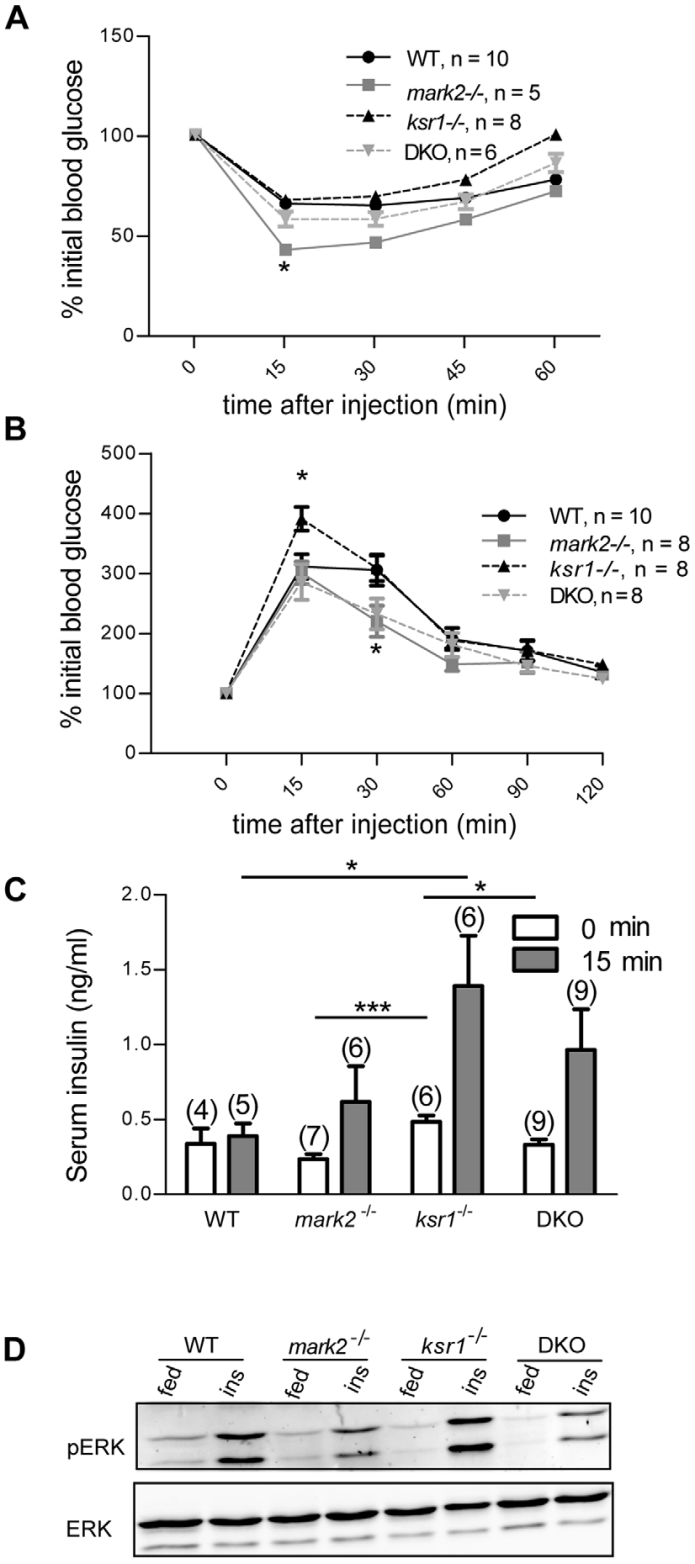
Deletion of KSR1 in *mark2^-/-^* mice reverts insulin sensitivity, but not glucose tolerance. A. Insulin tolerance tests (ITT) were performed on WT, *mark2^-/^* ^-^, *ksr1^-/-^* and DKO mice. Results shown are normalized to initial blood glucose levels. B. Glucose tolerance tests (GTT) were performed on WT, *mark2^-/^* ^-^, *ksr1^-/-^* and DKO mice. Results shown are normalized to initial blood glucose levels. C. Serum insulin levels before GTT (0 min) and 15 min after an IP injection of glucose (15 min). The number of mice analyzed under each condition is indicated above each bar. D. Insulin stimulated ERK activation in BAT. Mice were injected with insulin then sacrificed 15 min later. BAT was excised, lysed, and western blot performed using pERK and ERK specific antibodies. *p<0.05, ***p<0.001.

Insulin tolerance tests (ITT) were performed to determine the sensitivity of insulin-responsive tissues in the mice. *mark2* ^-/-^ mice display an increase in insulin sensitivity (Fig. 4A and [48]), while the peripheral tissues of *ksr1* ^-/-^ mice have insulin sensitivity similar to WT mice. DKO mice were compared to WT, *mark2* ^-/-^, and *ksr1* ^-/-^ mice for their ability to regulate glucose homeostasis. Disruption of KSR1 in *mark2* ^-/-^ mice reverses the enhanced insulin sensitivity resulting from MARK2 deletion giving DKO mice similar insulin sensitivity to that of WT and *ksr1^-/-^* mice. These insulin-regulation data suggest that MARK2 functions as a negative regulator of KSR1 in insulin sensitivity.

Glucose tolerance tests (GTT) showed that *ksr1* ^-/-^ mice have a slight, but significant, decrease in glucose tolerance at early time points. This is in contrast to *mark2* ^-/-^ mice, which have increased glucose tolerance (Fig. 4B and [48]). We found that deletion of KSR1 in *mark2* ^-/-^ mice does not revert the enhanced glucose tolerance observed in *mark2* ^-/-^ mice. Instead, DKO mice have glucose tolerance similar to *mark2* ^-/-^ mice. As glucose tolerance is a composite of the effects of glucose on insulin secretion and the responsiveness of peripheral tissues for insulin-stimulated glucose uptake, the results could reflect a combination of increased insulin responsiveness of peripheral tissues to *mark2* disruption and increased insulin secretion in response to *ksr1* deletion.

### Serum insulin levels in ksr1^-/-^ and mark2^-/-^ mice

MARK2 and KSR1 are both expressed in pancreatic islets. *mark2* ^-/-^ beta cells have altered polarity [49]. To test whether the effects of MARK2 and KSR1 on glucose tolerance is due to differences in insulin secretion, serum insulin levels were measured. Fasting serum insulin levels indicated that *ksr1* ^-/-^ mice have a higher level of basal insulin secretion compared to WT and *mark2* ^-/-^ mice (Fig. 4C). Fifteen minutes after a glucose load all genotypes showed comparable fold elevation in serum insulin, however, *ksr1* ^-/-^ mice had three-fold higher absolute serum insulin levels relative to WT mice. Importantly, after glucose stimulation, DKO mice display serum insulin levels that are intermediate between *mark2* ^-/-^ and *ksr1^-/-^* mice. These data are consistent with a combined effect of enhanced insulin release due to KSR1 disruption which is moderated by enhanced sensitivity due to loss of MARK2.

ERK is activated by insulin in BAT [50], and KSR1 is necessary for proper insulin-stimulated ERK activation in HIRcB fibroblast cells [51]. To determine if MARK2 is regulating the activation of ERK via regulation of KSR1, ERK activation in BAT of mice 15 minutes after insulin treatment was examined (Fig. 4D). ERK activation is decreased in mice lacking MARK2, with or without KSR1 present. This result indicates that MARK2 may be acting upstream of ERK in BAT. Interestingly, the deletion of KSR1 did not effect insulin-stimulated ERK activation in BAT (Fig. 4D).

## Discussion

Proteomic analys reveal that MARK2 interacts with KSR1 [44] and we have shown that MARK2, similar to C-TAK1, is able to phosphorylate KSR1 *in vitro* on S392. This phosphorylation site has been shown previously to be a negative regulatory site of KSR1 [18], [31]. This result predicts that MARK2 negatively regulates KSR1 as an ERK scaffold. However, it is also possible that KSR1 serves as a scaffold for MARK2 similar to the interaction of KSR1 with ERK [42], [52].

As the MARK family contains multiple members, it is possible that other members of the MARK family are able to compensate for the loss of MARK2. However, though the family has a high degree of homology, the subcellular localization varies. MARK1, MARK2, and C-TAK1 are all basolateral, but C-TAK1 is also found on the apical surface [9]–[12]. MARK4 does not display asymmetric localization, but interacts with filamentous structures [9]. The family members are also differentially regulated. MARK2 localizes to the cytoplasm upon overexpression of PKCζ [9]. However, MARK1 and C-TAK1 do not alter their localization when PKCζ is overexpressed [9]. This observation suggests that other members of the MARK family may not fully compensate for the loss of MARK2.

These data raise the possibility that different stimuli could selectively recruit related members of a kinase family to impair KSR1 function through phosphorylation of a common site. In this model, KSR1 would integrate different signals to the same effect. These mechanisms may allow KSR1 to respond to signals in different cell types or in multiple subcellular compartments. The potential of KSR1 to receive input at the same site from multiple kinases may also affect the intensity and/or duration of KSR1-mediated signaling by increasing the stoichiometry of KSR1 phosphorylation on Ser392.

To evaluate the interplay between KSR1 and MARK2 *in vivo*, we generated *ksr1^-/-^*, *mark2^-/-^*, and DKO mice. MARK2 is a negative regulator of insulin tolerance as *mark2^-/-^* mice show increased responsiveness to exogenous insulin (Fig. 4A). This is consistent with previous analysis of *mark2^-/-^* mice [26]. In contrast, disruption of KSR1 has no effect on insulin tolerance. However, the deletion of KSR1 in *mark2^-/-^* mice led to a reversal of the improved insulin tolerance of *mark2^-/-^* mice (Fig. 4A). These data suggest that KSR1 acts downstream of MARK2 to enhance insulin action in peripheral tissues.

In addition, we observed that disrupting KSR1 modestly impairs glucose tolerance. This is in contrast to MARK2 deletion, which mildly enhances glucose metabolism. When MARK2 and KSR1 are deleted together, DKO mice display an increase in glucose tolerance similar to *mark2^-/-^* mice, indicating that MARK2 is genetically downstream of KSR1 in glucose regulation. The inverted genetic relationship of KSR1 and MARK2 in insulin tolerance versus glucose tolerance may be indicative of the tissues in which they act. GTTs measure the combined effect of a glucose load on insulin secretion from pancreatic islets and insulin action on target tissues. ITTs measure the action of insulin only in target tissues. Our previous hyperinsulinemic/euglycemic clamp studies demonstrate that the peripheral tissues of *ksr1^-/-^* mice respond normally to insulin [47]. This suggests that the mild glucose intolerance of *ksr1^-/-^* mice reflects a role for KSR1 in insulin secretion. Consistent with this possibility, we observed increased serum insulin levels following a glucose challenge in *ksr1^-/-^* mice relative to WT mice. That DKO mice had a level of serum insulin intermediate between *ksr1^-/-^* and *mark2^-/-^* mice may reflect the consequence of combining increased insulin secretion following KSR1 disruption with a decreased demand for insulin in peripheral tissues lacking MARK2.

Deletion of MARK2 from BAT decreases the amount of pERK present after insulin treatment, indicating that MARK2 may act upstream of ERK signaling in response to insulin in BAT. The deletion of KSR1 did not effect insulin-stimulated ERK activation in BAT. This could be due to compensation by KSR2. KSR2, like KSR1, is able to regulate the Raf/MEK/ERK MAP kinase pathway [44]. KSR2 regulates glucose homeostasis, at least in part by regulation of AMPK, which is in the same family of kinases as MARK2 [47].

Perhaps the most striking biological effect of KSR1 disruption is the 3-fold greater level of serum insulin observed in comparison to WT mice after glucose administration (Fig. 4C). In combination with normal ITT (Fig. 4A) and insulin responsiveness of *ksr1^-/-^* mice following hyperinsulinemic euglycemic clamp [47], these data indicate an unexpected role for KSR1 in insulin secretion. Future studies should reveal the signaling mechanism regulated by KSR1 to moderate insulin secretion, determine whether its role is independent of its function as a scaffold for the Raf/MEK/ERK kinase cascade and assess the long-term effects of KSR1 disruption on beta cell function.
